# The impact of a hybrid social marketing intervention on inequities in access, ownership and use of insecticide-treated nets

**DOI:** 10.1186/1475-2875-6-13

**Published:** 2007-01-29

**Authors:** Sohail Agha, Ronan Van Rossem, Guy Stallworthy, Thankian Kusanthan

**Affiliations:** 1 Department of International Health and Development, School of Public Health and Tropical Medicine, Tulane University, New Orleans, USA; 2 Ghent University, Department of Sociology, Ghent, Belgium; 3 PSI/Myanmar, Kamaryut Township Yangon, Myanmar; 4 University of Zambia, Department of Gender Studies, Lusaka, Zambia

## Abstract

**Background:**

An ITN intervention was initiated in three predominantly rural districts of Eastern Province, Zambia, that lacked commercial distribution and communication infrastructures. Social marketing techniques were used for product and message development. Public sector clinics and village-based volunteers promoted and distributed subsidized ITNs priced at $2.5 per net. A study was conducted to assess the effects of the intervention on inequities in knowledge, access, ownership and use of ITNs.

**Methods:**

A post-test only quasi-experimental study design was used to compare intervention and comparison districts. A total of 2,986 respondents were interviewed. Survey respondents were grouped into four socio-economic (SES) categories: low, medium-low, medium and high. Knowledge, access, ownership and use indicators are compared. Concentration index scores are calculated. Interactions between intervention status and SES help determine how different SES groups benefited from the intervention.

**Results:**

Although overall use of nets remained relatively low, post-test data show that knowledge, access, ownership and use of mosquito nets was higher in intervention districts. A decline in SES inequity in access to nets occurred in intervention districts, resulting from a disproportionately greater increase in access among the low SES group. Declines in SES inequities in net ownership and use of nets were associated with the intervention. The largest increases in net ownership and use occurred among medium and high SES categories.

**Conclusion:**

Increasing access to nets among the poorest respondents in rural areas may not lead to increases in net use unless the price of nets is no longer a barrier to their purchase.

## Background

Widespread coverage of vulnerable populations with insecticide-treated nets is a critical component of the Roll Back Malaria strategy. ITN coverage remains low in Africa [1,2] with price being a major barrier to the use of ITNs [3-6]. Several subsidized approaches to net distribution have been used. However, evidence on how to target vulnerable groups remains limited outside of relief efforts and community based programmes. An important gap in knowledge is how ITN interventions influence socio-economic (SES) differences in access, ownership and use of nets [7,2].

Direct subsidy on products or indirect subsidies via vouchers have been used to target low-income, vulnerable populations through ITN interventions. Direct partial subsidies may be provided through social marketing programmes, which generate demand for ITNs through advertising and promotion and distribute ITNs through retail outlets or sales agents. While social marketing ITN interventions have been associated with increases in ITN use, the degree to which they are able to reach low SES groups merits further examination.

An assessment of the performance of a social marketing intervention in Tanzania, where nets were priced at $5.4 per net, showed that net ownership increased substantially but that the SES gradient in net ownership was steep and nets did not reach the poorest households [4]. An assessment in Kenya showed that access to nets through the retail sector disadvantaged remote communities where mothers were less well educated [8]. During its first two years, a social marketing programme in Blantyre district, Malawi, marketed a blue conical net in urban areas at $6.55 and a green rectangular net in rural areas at $4.33. A survey conducted 15 months after the social marketing programme showed that while net use increased substantially, significant differentials emerged in the use of nets by socio-economic status and urban/rural residence [9]. Subsequently, the Malawi social marketing intervention expanded nationally and sold heavily subsidized nets at $0.6 to penetrate rural areas. This led to the intervention achieving very high sales volumes [10]. A comparison of nationally representative Demographic and Health Surveys data shows that urban-rural and socio-economic differentials in net use declined after the heavily subsidized net was introduced [11,12]. Another assessment of a social marketing intervention in 25 villages in Tanzania showed that, in the presence of an extremely active commercial sector for nets, social marketing of nets at a retail price of $5 was associated with an increased equity in ownership of nets [13].

This study assesses the effects of a hybrid social marketing intervention to increase knowledge, access and use of ITNs in a primarily rural province of Zambia. Unlike other social marketing interventions that have relied primarily on the retail sector for net distribution [8], this intervention is considered a hybrid because it combines a social marketing approach with distribution of nets through public health facilities. Nets were sold through rural health centers for $2.5 per net. This study asks three main questions. Was there an overall increase in knowledge, access, ownership and use of ITNs? Were there declines in socio-economic inequities in these outcomes? How did different socio-economic groups benefit from the intervention?

### Social marketing in a rural context

#### The commercial infrastructure

More than 90% of the residents of the Eastern province of Zambia live in rural areas. Basic road infrastructure in this province is poor and many parts of the province become inaccessible during the rainy season. The commercial distribution system is very limited. Wholesale distribution of products is limited to Chipata city, the provincial headquarters, and to one other district in Eastern province – Katete. Shopkeepers from larger villages travel to Chipata or Katete to purchase fast moving consumer products such as soaps, detergents, cooking oil, sugar and maize meal. Due to lack of public transportation, shopkeepers usually travel on their bicycles to Chipata or Katete and bring back products on their bicycle carriers. There is no system for the movement of goods to smaller villages in Eastern province. In order to obtain consumer products, residents of smaller villages have to travel for several hours to the nearest large village. Mass media reach is low in Eastern province: nearly seven out of 10 adult women in Eastern province have no access to any form of mass media; about 20% of women listen to radio every day and 3% watch television every week [14]. Because of the limited development of a commercial infrastructure in Eastern province, the social marketing programme relied on a partnership with the public sector for the distribution and promotion of nets.

#### The use of government clinics and village-based promoters

The ITN intervention was implemented through government health clinics in three districts of Eastern province: Chipata, Lundazi and Chama. Nets were sold and promoted through government health clinics, managed by the District Health Management Teams (DHMTs). There are a total of 64 health clinics in these three districts. Staff at the majority of these clinics were trained to provide residents with information on the use of ITNs and to motivate them to purchase and use ITNs. By the time this survey was conducted, one staff member at about half the government clinics in Chipata and Lundazi and all the health clinics in Chama was trained to promote the use of ITNs.

Because government health clinics are relatively few in number and their geographic distribution is often not according to the size or the needs of rural Zambian communities [15], it was deemed necessary to have an additional mechanism for the promotion and distribution of nets. The project established neighbourhood health committees to promote the use of ITNs. Each neighbourhood health committee included a volunteer ITN promoter living in the village. Approximately 600 village-based volunteers were trained to provide information on nets and to motivate village residents to purchase and use ITNs. Hence, the intervention relied on public sector clinics and village-based promoters.

The intervention was designed to remove obstacles to behaviour change through interpersonal communication. It aimed to remove misconceptions about the causes of malaria and to educate local residents about ways of preventing malaria. For example, many Zambians believe that malaria is caused by cold or changing weather, by drinking and eating dirty water and cold food [16] or by supernatural agents [17]. Commonly used household strategies to prevent malaria in Zambia include smoking the house or sitting area with local herbs, managing the environment through cutting grass, draining or filling holes that contain water, closing doors and windows during the rainy season and avoiding cold food [15,17]. The intervention sought to convince residents of Chipata, Lundazi and Chama districts of the effectiveness of treated nets as a way of preventing malaria and made less expensive nets widely available at the clinic and village levels.

Prior to the start of the intervention, the lack of availability of nets in rural Zambia was a major obstacle to their use. One qualitative study of 240 households in Eastern province found that 7% of households owned mosquito nets [17]. The commercial availability of nets and insecticide was very limited. When available, nets were sold at high prices. In the absence of a major local producer of nets in Zambia, several commercial companies market imported nets. However, these nets are sold for about US $27 per net and were unaffordable for most Zambians [15]. About 73% of Zambian households fall below the poverty line, having less or up to US$1 per day.

As part of the social marketing intervention, nets were sold through rural health centers at $2.5 per net. Because small-scale farmers have cash income only available during certain times of the year, net purchasers could obtain nets on a partial down payment – after agreeing to a payment schedule. The nets marketed for this intervention were untreated 100% polyester double and family size green nets that were branded as POWERNET. Each net was packaged with a K-O Tab home insecticide treatment kit that was branded as POWERCHEM. The implementation of the ITN intervention began in September 1998.

## Methods

### Study design

A quasi-experimental study design was used for this assessment. Three intervention and two comparison districts were compared on a post-intervention measurement. A potential problem for such a design is the lack of a pre-intervention measurement, which leaves open the possibility that observed differences between control and intervention groups are due to pre-existing differences.

The data used in this evaluation is from a household survey implemented in 5 out of 8 districts of Eastern province, Zambia. The purpose of the survey was to provide data for a mid-term assessment of the ITN intervention in Eastern Province. The ITN intervention in Eastern Province started in 1998. The intervention districts were Chipata (pop. 362,132), Lundazi (pop. 236,732) and Chama (pop. 76,685). They were chosen because they had stronger sentinel surveillance systems than the other districts. The control districts were Chadiza (pop. 82,400) and Petauke (pop. 242,533). The control districts were selected because data from Zambia's Central Statistical Office showed that socio-economic conditions in these two districts were similar to those in the three intervention districts. In total, these five districts had a combined population of approximately one million people, or about 10% of the population of Zambia [18].

### Sampling

Respondents were selected using a four stage stratified cluster sampling procedure implemented by the Central Statistical Office. At the first stage, districts were selected purposively. At the second stage, 150 Statistical Enumeration Areas (SEAs) were randomly selected using a probability proportional to size scheme for urban and rural areas within each district. At the third stage, households were selected using interval-sampling within each SEA. At the fourth stage, one eligible member in each household was randomly selected for participation in the survey. To be eligible for the study a household member had to be between 15 and 49 years of age, and to have at least one child under five years of age. The survey planned to interview 600 respondents in each district. The total number of interviews completed in the five districts was 2,986.

### Questionnaire development

The questionnaire used was developed after a review of the literature on ITN use in Africa and questionnaires used in other ITN surveys implemented in Africa. The questionnaire was pre-tested in Lusaka prior to being fielded. It was translated into the two most widely spoken languages Zambian languages, Bemba and Nyanja. The data collection was carried out between August and September 2000 by a team of 16 trained interviewers and four supervisors. The refusal rate was 1.2 percent.

### Variables

#### Intervention status

Intervention status was operationalized as a dichotomous variable with a value of 1 for respondents in the intervention districts and a 0 for respondents in the comparison districts.

#### Socio-economic status

The socio-economic status of the household was measured using an index of assets. This index was calculating by counting the number of household assets owned (bicycle, motorcycle, car, stove, video player, radio, television, farm, house, refrigerator or deep freezer) and amenities/services used (telephone, electricity, piped water in the home or plot, own flush toilet, cement or tile floor). The range for this index is 0 to 15. A categorical assets index variable was constructed by recoding the scores in approximately quartiles. In the sample the four assets index categories were: low (score: 0–1), medium-low (score: 2), medium (score: 3), and high (score: 4–15).

#### Socio-demographic variables

These included the urban/rural residence, age of the respondent (in years), sex (0: female, 1: male), the number of children in the household, marital status of the respondent (1: married or cohabitating, 2: widowed, divorced or separated, 3: single) and educational level of the respondent (no schooling, junior primary, senior primary, junior secondary, senior secondary or higher).

#### Knowledge of malaria

Respondents were asked about their knowledge of malaria, their knowledge of the causes of malaria, and the symptoms and consequences/complications of malaria. As knowledge of malaria was nearly universal, this variable was not included in the analysis. With regard to the causes of malaria the study examined whether respondents mentioned mosquito bites as the cause of malaria.

#### Knowledge of mosquito nets and insecticide treatment

Knowledge of means of protection is measured in four general areas: knowledge of mosquito nets, knowledge of insecticide-treated mosquito nets (ITN), knowledge of PowerNET, and knowledge of POWERCHEM. Questions about mosquito nets included whether respondents were familiar with mosquito nets. Questions were asked about the insecticide treatment of mosquito nets. Respondents were asked if they had heard of treating nets with insecticide, and if they knew why nets were treated with insecticide. Respondents were given a score of 1 if they mentioned that the purpose of insecticide treatment was to kill mosquitoes and a 0 otherwise. The questionnaire also asked about the benefits of sleeping under an ITN. Respondents who answered that ITNs kills/repels mosquitoes or keep mosquitoes away were given a score of 1, and a 0 otherwise. Respondents were also asked about the disadvantages of sleeping under an ITN. Those who responded that there were no disadvantages were given a score of 1 on this variable, while others were given a score of 0. Respondents were asked if they knew that ITNs should be retreated regularly (at least every year or after 3 washes). A final set of questions focused on respondents' familiarity with the products marketed by SFH, the ITN PowerNET and the retreatment kit POWERCHEM. Respondents were asked if they had ever heard of these products. For POWERCHEM, respondents were asked whether they knew what it was. The answer that POWERCHEM is an insecticide to treat mosquito nets earned them a score 1 on this variable, while others received a score of 0.

#### Access to mosquito nets

Access to mosquito nets was measured by asking respondents how long it would take them to reach a nearby place where they could purchase a mosquito net. Two variables were created to measure access. The first access variable measured the number of minutes a respondent would take to reach a place where they could purchase a mosquito net, while the second access variable measured whether or not a respondent could reach a place to obtain a mosquito net within fifteen minutes. Respondents who reported not being able to obtain a mosquito net, were given a time of 10 hours and 1 minute, i.e., 1 minute more than the maximum reported time.

#### Beliefs about malaria protection

Respondents were asked if they believed that a person could protect themselves against malaria. Those who answered in the affirmative were asked how a person could protect themselves against malaria. Multiple responses were possible including sleeping under a mosquito net, sleeping under an ITN, keeping surroundings clean etc.

#### Behaviour

The behaviour variables refer to the respondents' possession and use of mosquito nets and ITNs, and purchase of PowerNETs and POWERCHEM in particular.

Respondents' behaviour with regard to ITN use was measured by the number of ITNs in the household. Other behavioural indicators measure if the respondent or anyone else in the household had ever purchased a PowerNET, if the respondent or anyone else in the household had ever purchased a POWERCHEM kit. Behavioural indicators also included the number of mosquito nets in the household and if the respondent usually slept under a mosquito net.

### Statistical analysis

#### Hypothesis 1: The intervention increased knowledge, access and the use of nets

Tests were conducted to measure differences between intervention and comparison districts in knowledge of, access to and use of nets. To control for socio-demographic differences between the comparison and intervention districts, logistic regression was used for the dichotomous outcomes and linear regression for the continuous outcomes. The results were presented as adjusted proportions for dichotomous outcome variables and adjusted means for ordinal and parametric variables. Adjusted proportions were calculated by substituting the sample means of socio-demographic variables for individual values of these variables in the logistic regression equation. Adjusted means were calculated in a similar manner, using OLS regression. The socio-demographic control variables included urban/rural residence, age, sex, number of children, marital status, education, and the number of assets. To compare adjusted proportions of the comparison and intervention groups, a likelihood ratio χ^2 ^test was used with 1 df. To compare adjusted means, *F*-tests were used with 1 and *N*-2 df. Unadjusted means were also compared on all outcome variables.

#### Hypothesis 2: The intervention contributed to declines in SES inequity in knowledge, access and use of nets

The programme impact on equity is assessed using a Concentration Index that calculates the degree of socio-economic inequity on the outcome variables. This index is preferred over other commonly used indices, such as the Gini coefficient or the inequality ratio (the ratio between the lowest and the highest income groups) because it has the ability to capture the experience of whole population [19]. The value of the concentration index theoretically ranges between -1 and +1. It has a value of 0 when there is no inequality. A positive value of the concentration index indicates inequality in favour of the wealthy, and a negative value suggests the opposite. Since the hypothesis is that the outcome inequality in the intervention group will be lower than in the comparison group, a one-tailed *t*-test is used to determine whether the concentration indices are significantly different from each other – i.e. whether there are significant differences in socio-economic inequity on outcomes between intervention and comparison districts.

#### Hypothesis 3: The intervention benefited SES groups differentially

Adjusted proportions and means were also used to assess which socio-economic groups benefited most from the intervention. Adjusted means or proportions were calculated for a combination of the four socio-economic categories (low, medium-low, medium and high) and the two groups (intervention and comparison) – i.e. for 8 categories. The primary interest was in testing whether there was an interaction between intervention status and socio-economic status. To test this interaction effect for each outcome, *F *tests with 3 and *N*-8 df and χ^2^-tests with 3 df were used for adjusted means and proportions respectively. A Type I error of 0.050 was considered acceptable for this analysis. A Bonferroni correction was used in the case of multiple comparisons, and α_b _= 0.050/*n*_*t *_where α_b _is the Type I error permitted after the Bonferroni correction, and *n*_*t *_the number of tests in the set. For example, for the cluster of 7 outcome variables under "knowledge of mosquito nets and treatment", the α_b _= 0.050/7 = 0.007. Hence, p-values larger than 0.007 will not be considered significant for individual interaction tests for outcome variables in this cluster.

## Results

### Sample description

Table 1 compares respondents in the comparison (column 1) and intervention (column 2) districts in terms of their socio-demographic characteristics. Independent sample *t*-tests were used to compare parametric or dichotomous variables in comparison and intervention districts and χ^2 ^tests were used for categorical variables. *P*-values from statistical tests are shown in column 4.

**Table 1:** Characteristics of respondents in comparison and intervention districts

	(1) Comparison (n = 1186)	(2) Intervention (n = 1800)	(3) Test statistic^a^	(4) *p*
Age of respondent	30.1	29.9	0.74	0.461
Number of children in household	3.1	3.1	-0.33	0.743
Number of children under 5 in household	1.7	1.7	-0.12	0.904
Number of children 5–14 in household	1.4	1.4	-0.29	0.770
Marital status				
Married	85.8%	87.9%	5.88^b^	0.118
Cohabiting	0.3%	0.3%		
Widowed/divorced/separated	9.6%	7.2%		
Single	4.3%	4.6%		
Sex: Male	26.7%	28.7%	-1.19	0.233
Residence: Rural	82.2%	76.1%	4.07	0.000
Asset index	2.6	2.9	-3.48	0.001
Assets				
Low (0–1)	30.6%	27.3%	25.41^b^	0.000
Medium low (2)	29.0%	24.1%		
Medium (3)	19.7%	20.5%		
High (4–15)	20.7%	28.1%		
Number of years of schooling completed	4.4	5.6	-8.77	0.000
Schooling				
No school	34.5%	20.6%	84.80^b^	0.000
Junior primary	16.5%	15.1%		
Senior primary	29.1%	35.9%		
Junior secondary	11.0%	16.4%		
Senior secondary or higher	8.9%	12.0%		

^a ^All tests for the differences between control and intervention districts are independent sample *t*-tests unless specified otherwise. ^b^χ^2^-tests.

Table 1 shows that there were no significant differences between comparison and intervention districts in mean age of respondents (30 years), mean number of children (3.1), mean number of children under five (1.7), mean number of children between five and fourteen (1.4), the proportion married (87%) and the proportion male (28%). The sample reflects the predominance of female-headed households in these rural districts of Zambia.

However, there were significant differences between comparison and intervention districts with regard to rural residence, socio-economic status (measured by an index of assets and the proportion of households with different asset levels) and education (measured by the number of years of schooling and the proportion of respondents with different educational levels): a larger proportion of respondents in comparison than in intervention districts (82% vs 76%) lived in rural areas; households in the comparison districts owned an average of 2.6 assets, compared to an average of 2.9 assets owned by households in intervention districts; 21% of respondents in comparison districts compared to 28% of respondents in intervention districts were from households in the high asset category (4–15); respondents in the comparison districts had completed fewer years of schooling than respondents in intervention districts (4.4 vs. 5.6); 35% of respondents in comparison districts compared to 21% in intervention districts had no schooling.

### Was there an overall increase in knowledge, access and use of nets?

Table 2 compares respondents in the comparison districts with respondents in the intervention districts on knowledge of and access, to nets and beliefs and behaviour related to malaria prevention. The table shows mean values on outcomes after adjusting for age, sex, urban/rural residence, education, marital status, number of children and assets. Column 1 and 2 show mean values on outcomes in comparison and intervention districts, respectively. Column 4 shows *p*-values from the statistical tests.

**Table 2:** Adjusted^a ^estimates for mean scores on outcome variables for comparison and intervention districts

	(1) Comparison	(2) Intervention	(3) Test statistic^c^	(4) *p*
**Knowledge of malaria**				
Mosquito bites cause malaria	58.7%	76.1%	86.15	0.000
**Knowledge of nets and treatment**				
Familiar with mosquito nets	92.0%	95.9%	27.35	0.000
Ever heard of insecticide treatment	14.4%	51.9%	409.08	0.000
ITN kills or repels mosquitoes	10.7%	39.4%	302.32	0.000
Know that ITN needs re-treatment	2.3%	19.3%	245.06	0.000
No disadvantages to ITN use	7.5%	24.2	150.35	0.000
Ever heard of PowerNET	10.8%	69.4%	897.58	0.000
Ever heard of POWERCHEM	1.9%	20.7%	319.71	0.000
**Access to mosquito nets**				
Access to mosquito nets (in minutes)	222.0	131.4	136.20^d^	0.000
Access to nets in 15 minutes or less	8.8%	28.1%	171.14	0.000
**Beliefs about malaria protection**				
One can protect oneself from malaria	57.8%	75.7%	94.57	0.000
One can protect by using a net	18.1%	37.2%	116.24	0.000
One can protect by using an ITN	1.0%	8.0%	108.59	0.000
**Behaviour**				
Number of mosquito nets in household	0.26	0.41	36.07^d^	0.000
Number of ITNs owned by household	0.06	0.25	117.14^d^	0.000
Ever had ITN in household	1.3%	14.0%	225.44	0.000
Ever bought PowerNET	1.4%	14.6%	248.55	0.000
Ever bought POWERCHEM	0.4%	5.1%	100.51	0.000
Usually sleeps under a net	5.4%	13.3%	62.52	0.000

^a^Means and proportions adjusted for age of respondents, assets, sex, urban/rural residence, education, marital status and number of children. ^b^Not adjusted for marital status. ^c^χ^2^-test with1 df, unless specified otherwise. ^d^*F*-test with 1 & 2974 df.

A larger proportion of respondents in the intervention districts (76%) than in the comparison districts (59%) were aware that mosquito bites cause malaria (p = 0.000). Although most respondents in intervention (96%) and comparison districts (92%) were familiar with mosquito nets, the percentage was higher in the intervention area.

Knowledge about insecticide treatment and of ITNs was considerably higher among intervention than in comparison respondents: 52% of respondents in the intervention compared to 14% in the comparison districts had heard of insecticide treatment; 39% of intervention and 11% of comparison respondents knew that ITNs kill or repel mosquitoes; 19% of intervention respondents and 2% of comparison respondents knew that ITNs need re-treatment; 24% of respondents in the intervention districts and 8% in the comparison districts felt that there were no disadvantages of using an ITN. Knowledge about PowerNET and POWERCHEM brands was also substantially higher among intervention than in comparison respondents: 69% of intervention and 11% of comparison respondents had heard of PowerNET; 21% of intervention and 2% of comparison respondents had heard of POWERCHEM.

Access to mosquito nets was higher in intervention than in comparison districts: on average, intervention district respondents reported that it would take them 131 minutes to obtain a mosquito net whereas comparison district respondents reported that it would take them 222 minutes to obtain a mosquito net; 28% of intervention and 9% of comparison district respondents reported that it would take them 15 minutes or less to obtain a mosquito net.

Self efficacy regarding protection from malaria was higher among intervention district respondents: 76% of respondents in intervention and 58% in comparison districts reported that one can protect oneself from malaria; 37% of intervention and 18% of comparison respondents reported that one can protect oneself from malaria by using a mosquito net; 8% of respondents in intervention and 1% in comparison districts reported that one can protect oneself from malaria by using a treated mosquito net.

The average number of mosquito nets in the household was higher in the intervention (0.41) than in the comparison (0.26) districts. Intervention district respondents also reported a higher number of ITNs (0.25 vs 0.06) and a higher number of POWERNETS (0.29 vs 0.07) than comparison district respondents. The proportion who reported that they had an ITN in the household (14% vs 1%) and had ever bought POWERNET (15% vs 1%) was also higher in the intervention than in the comparison districts. About 5% of respondents in intervention and less than 1% in comparison districts reported that they had bought POWERCHEM. Of those who had treated a mosquito net (n = 390), 79.0% reported to have used POWERCHEM the last time, 4.6% KO tab, 2.3% another brand, while 14.1% could not remember the insecticide brand used (not shown).

Consistent with higher rates of ownership, the use of mosquito nets was higher in the intervention districts: 13% of intervention and 5% of comparison respondents reported that the usually sleep under a mosquito net.

Ninety two percent of respondents gave their inability to afford a net as the main reason for not using a mosquito net (not shown). Respondents who had not treated mosquito nets (n = 759) gave a variety of reasons for not having done so: 24% mentioned that they could not afford insecticide treatment, 13.0% reported that they did not know how to treat a mosquito net, 5% did not know where to buy the treatment kits from, 4% reported that insecticide was not available and 13% reported that they had not yet treated their nets because the mosquito net had not been washed 3 times or it had not been owned it for a year (not shown).

### Did socio-economic inequity in knowledge, access and use of nets decline?

Table 3 compares socio-economic inequity on the outcome variables in the comparison and the intervention districts, using the difference in scores on the concentration index. A higher score on the index shows greater socio-economic inequity. The first column of Table 3 shows the concentration index scores on outcomes in the comparison districts. The second column shows concentration index scores on outcomes in the intervention districts. Columns 3, 4 and 5 show the magnitude of the difference in concentration index scores between intervention and comparison districts, the t-test statistic to determine whether concentration indices are different from each other and the p-value associated with the t-test, respectively. To illustrate, the concentration index score on the outcome mosquito bites cause malaria is higher in comparison (0.129) than in intervention (0.069) districts and the difference in concentration index scores (0.059) is statistically significant (p = 0.009). This shows that there is less socio-economic inequity in knowledge of malaria in intervention districts.

**Table 3:** Concentration indexes for comparison and intervention districts

	(1) Comparison	(2) Intervention	(3) Difference	(4) *t*^a^	(5) *p*
**Knowledge of malaria**					
Mosquito bites cause malaria	0.129	0.069	0.059	3.51	0.009
**Knowledge of nets and treatment**					
Familiar with mosquito nets	0.050	0.013	0.037	5.03	0.002
Ever heard of insecticide treatment	0.366	0.131	0.235	6.54	0.001
ITN kills or repels mosquitoes	0.376	0.129	0.247	5.80	0.002
Know that ITN needs re-treatment	0.675	0.182	0.493	8.92	0.000
No disadvantages to ITN	0.388	0.144	0.243	4.73	0.005
Ever heard of PowerNET	0.483	0.084	0.399	10.92	0.001
Ever heard of POWERCHEM	0.702	0.246	0.455	10.59	0.000
**Access to mosquito nets**					
Access to mosquito nets (in minutes)	-0.151	-0.132	-0.019	-0.77	0.768
Access to nets in 15 minutes or less	0.423	0.166	0.257	5.28	0.003
**Beliefs about malaria protection**					
One can protect oneself from malaria	0.108	0.068	0.041	2.41	0.030
One can protect by using a net	0.332	0.126	0.205	5.76	0.001
One can protect by using an ITN	0.549	0.279	0.270	2.23	0.056
**Behaviour**					
Number of mosquito nets in household	0.637	0.434	0.203	7.05	0.000
Number of ITNs owned by household	0.726	0.400	0.326	8.43	0.000
Ever had ITN in household	0.709	0.339	0.370	8.95	0.000
Ever bought PowerNET	0.721	0.359	0.362	9.75	0.000
Ever bought POWERCHEM	0.749	0.399	0.350	6.52	0.000
Usually sleeps under a net	0.608	0.374	0.234	5.80	0.001

^a^: *t*-statistic with df=5(sc2+si2)2(sc2)2+(si2)2−2
 MathType@MTEF@5@5@+=feaafiart1ev1aaatCvAUfKttLearuWrP9MDH5MBPbIqV92AaeXatLxBI9gBaebbnrfifHhDYfgasaacH8akY=wiFfYdH8Gipec8Eeeu0xXdbba9frFj0=OqFfea0dXdd9vqai=hGuQ8kuc9pgc9s8qqaq=dirpe0xb9q8qiLsFr0=vr0=vr0dc8meaabaqaciaacaGaaeqabaqabeGadaaakeaacqWGKbazcqWGMbGzcqGH9aqpdaWcaaqaaiabiwda1maabmaabaGaem4Cam3aa0baaSqaaiabdogaJbqaaiabikdaYaaakiabgUcaRiabdohaZnaaDaaaleaacqWGPbqAaeaacqaIYaGmaaaakiaawIcacaGLPaaadaahaaWcbeqaaiabikdaYaaaaOqaamaabmaabaGaem4Cam3aa0baaSqaaiabdogaJbqaaiabikdaYaaaaOGaayjkaiaawMcaamaaCaaaleqabaGaeGOmaidaaOGaey4kaSYaaeWaaeaacqWGZbWCdaqhaaWcbaGaemyAaKgabaGaeGOmaidaaaGccaGLOaGaayzkaaWaaWbaaSqabeaacqaIYaGmaaaaaOGaeyOeI0IaeGOmaidaaa@4CCD@, one-tailed.

In the comparison districts, the magnitude of the scores on outcomes related to insecticide treatment range from 0.366 to 0.732: there is substantial inequity in having ever heard of insecticide treatment (0.366), knowing that ITNs kill or repel mosquitoes (0.376), knowing that ITNs need retreatment (0.675), having heard of PowerNET (0.483) and having heard or POWERCHEM is (0.702). In the intervention districts, scores on these outcomes range from 0.084 to 0.260, indicating lower levels of socio-economic inequity in having heard of insecticide treatment (0.131), knowing that ITNs kill or repel mosquitoes (0.129), knowing that ITNs need retreatment (0.182), having heard of PowerNET (0.084) and having heard of POWERCHEM is (0.246). P-values show that the differences between concentration index scores in intervention and comparison areas are statistically significant.

Intervention districts have lower inequity in access to mosquito nets within 15 minutes: the concentration index score on the outcome of access within 15 minutes is 0.423 in comparison and 0.166 in intervention districts and the difference in scores is statistically significant (*p *= 0.003). Socio-economic inequity in the beliefs that one can protect oneself from malaria (0.108 vs. 0.068, *p *= 0.030) and one can protect oneself from malaria by using a mosquito net (0.332 vs. 0.126, *p *= 0.001) is lower in the intervention districts.

Socio-economic inequity in behavioural outcomes is generally of a larger magnitude than inequity in knowledge, showing larger differences in behavior than in knowledge: concentration index scores on behavioural outcomes range from 0.608 to 0.749 in comparison districts and from 0.339 to 0.434 in intervention districts. Concentration index scores in intervention districts are significantly lower than scores in comparison districts on all behavioural outcomes: there is significantly lower socio-economic inequity in the number of mosquito nets and the number of insecticide treated mosquito nets in the intervention districts; inequity in the purchase of POWERNET and POWERCHEM and in usually sleeping under a net is also lower in intervention districts.

### Did the benefits of the intervention accrue equally among socio-economic groups?

The earlier analyses show that the ITN intervention had desirable overall effects on knowledge, access and behaviours related to malaria prevention. In addition, socio-economic inequity in knowledge, access and behaviour declined in intervention districts. Since declines in inequity may occur due to proportionate improvements in all socio-economic groups or due to disproportionate improvements in lower socio-economic groups, the study examines how the benefits of the intervention were distributed among socio-economic groups.

Columns 1–8 in Table 4 shows the adjusted proportions and means on the outcome variables for a combination of the four SES and the two intervention status categories (i.e. a total of 8 categories) and columns 9 and 10 show the results of the test for the interaction between SES and intervention status. A significant p-value indicates that the intervention had differential effects on outcome variables by SES. However, the interaction does not indicate the particular SES category that the intervention had greatest effect on. To determine which SES group benefited most from the intervention, the net difference between the comparison and the intervention group within a socio-economic status category is compared to the net difference between the comparison and the intervention group in another socio-economic status category. To make the tests more conservative, a Bonferroni correction is used to determine statistical significance levels for a cluster of variables.

**Table 4:** Intervention effects by socio-economic status of respondents

M^a^	Low (0–1)		Medium-low (2)		Medium (3)		High (4+)		Test for interactions^c^	
	(1) Comp. (n = 363)	(2) Inter. (n = 492)	(3) Comp. (n = 344)	(4) Inter. (n = 433)	(5) Comp. (n = 234)	(6) Inter. (n = 369)	(7) Comp. (n = 245)	(8) Inter. (n = 506)	(9) Test statistic	(10) *p*

**Knowledge of malaria**										
Mosquito bites cause malaria	52.0%	71.1%	56.7%	73.9%	55.4%	72.0%	69.5%	85.3%	0.60	0.896
**Knowledge of nets and treatment**										
Familiar with mosquito nets	86.3%	95.0%	92.6%	95.3%	94.5%	95.6%	95.3%	96.8%	6.92	0.074
Ever heard of insecticide treatment	6.9%	42.2%	11.6%	47.9%	17.7%	56.4%	25.7%	63.2%	5.63	0.131
ITN kills or repels mosquitoes	4.6%	32.5%	8.4%	38.2%	12.9%	43.4%	19.2%	47.0%	10.25	0.017
Know that ITN needs re-treatment	0.3%	14.4%	0.0%	19.7%	2.3%	20.9%	7.2%	26.3%	29.94	0.000
No disadvantages to ITN	3.1%	17.3%	4.9%	25.6%	11.0%	31.6%	13.8%	27.8%	12.22	0.007
Ever heard of PowerNET	4.9%	63.7%	6.4%	65.8%	9.7%	69.2%	25.0%	76.4%	18.84	0.000
Ever heard of POWERCHEM	0.0%	16.0%	0.6%	19.0%	1.4%	23.3%	5.0%	29.4%	16.22	0.001
**Access to mosquito nets**										
Access to mosquito nets (in minutes)	282.76	132.49	216.73	138.10	198.85	147.47	171.93	107.90	8.85	0.000
Access to nets in 15 minutes or less	4.4%	26.4%	5.6%	22.4%	9.4%	28.5%	17.7%	38.1%	9.80	0.020
**Beliefs about malaria protection**										
One can protect oneself from malaria	52.6%	69.3%	53.1%	72.8%	61.7%	77.6%	64.2%	83.5%	1.86	0.601
One can protect by using a net	11.4%	34.9%	12.8%	34.8%	20.5%	35.8%	30.6%	44.9%	12.98	0.005
One can protect by using an ITN	0.4%	5.1%	0.6%	7.6%	0.4%	11.2%	2.2%	11.1%	4.84	0.184
**Behaviour**										
Number of mosquito nets in household	0.15	0.16	0.10	0.23	0.22	0.42	0.57	0.88	6.86	0.000
Number of ITNs owned by household	0.07	0.10	0.03	0.15	0.04	0.27	0.05	0.51	27.48	0.000
Ever had ITN in household	0.0%	6.8%	0.0%	12.0%	2.0%	21.0%	4.3%	26.8%	12.25	0.007
Ever bought PowerNET	0.0%	6.8%	0.0%	12.3%	2.0%	22.2%	4.6%	29.5%	12.22	0.007
Ever bought POWERCHEM	0.0%	1.9%	0.0%	4.6%	0.4%	9.4%	1.7%	11.1%	5.66	0.129
Usually sleeps under a net	1.1%	6.9%	2.1%	9.5%	7.6%	20.2%	17.9%	30.4%	7.76	0.051

^a ^Means and proportions adjusted for age of respondent, sex of respondent, urban/rural residence; education, marital status, and number of children. ^b ^Not adjusted for marital status (divorced/separated/windowed). ^c ^Test statistic depends on whether adjusted mean or adjusted proportion was estimated: for adjusted means the test statistics used an *F*-test was used with 7 and 2971 df for the between group differences and with 3 and 2971 df for the interaction effects, for adjusted proportions a likelihood ratio test was used following a χ^2 ^distribution with 7 df for the between group differences and 3 df for the interaction effect.

Several variables showed significant interactions between intervention status and SES. After a Bonferroni correction (α_b _= 0.050/7 = 0.007), 3 of the 7 variables representing knowledge of nets and insecticide treatment showed significant interaction terms. There was an interaction between knowledge that ITNs need retreatment and intervention status (*p *= 0.000). The net difference of 14% between comparison (0.3%) and intervention (14.4%) groups on this outcome was smaller in the low SES category than in other SES categories (which had net differences of about 19%). For the outcome ever having heard of POWERCHEM (*p *= 0.001), the intervention effect was also smaller in the low SES category (16%) compared to other SES categories (over 19%). For the outcome ever having heard of POWERNET (*p *= 0.000), the effect of the intervention was smaller in the high SES category (51%) compared to other categories (about 60%). This shows that the effect of the intervention in terms of improving knowledge of nets and treatment was not consistently stronger for any particular SES category..

For access to mosquito nets, the effect of the intervention in increasing access to mosquito nets was strongest in the low SES category. The decrease in the mean time required to obtain a mosquito net was greater for the low SES category than for other SES categories; respondents in the low SES category in the intervention districts needed an average of 150 fewer minutes to obtain a mosquito net than those living in the comparison districts (133 vs. 283 minutes); respondents in the medium-low, medium and high SES categories in the intervention districts needed between 50 and 80 fewer minutes to obtain a mosquito net than respondents in comparison districts.

After a Bonferroni correction, one of the three indicators reflecting beliefs about malaria (α_b _= 0.050/3 = 0.016) had significant interactions. The effects of the intervention on the belief that one can protect oneself from malaria by using a mosquito net (*p *= 0.005) were stronger in the low and medium-low SES categories.

After a Bonferroni correction (α_b _= 0.050/6 = 0.008), there were significant interactions between intervention status and SES for four out of six behavioural outcomes. Indicators reflecting ownership and purchase of ITNs as well as use of mosquito nets showed that the effects of the intervention were stronger in the medium-low, medium and high SES categories compared to the low SES category. Little change occurred in ownership, purchase and use of nets among the poorest respondents.

## Discussion

This study illustrates how the implementation of an ITN intervention affected knowledge, access and use of ITNs in rural Zambia, in a relatively isolated area where resources for the implementation of a social marketing intervention through retail outlets were very limited. The social marketing intervention partnered with the public sector in order to promote behavior change and promote subsidized nets to rural Zambians.

Although the cross-sectional study design does not permit causal attribution, findings indicate that respondents in intervention areas had higher awareness of insecticide treatment and understood why nets were treated with insecticide and required re-treatment. Respondents in intervention districts had higher access to mosquito nets than respondents in comparison districts. Consistent with improvements in knowledge, access and self-efficacy, increases in ITN ownership and use were associated with the intervention.

In spite of a substantial subsidy and a mechanism for making net purchase easier for rural residents (such as payment in installments), however, there was little change in net use among respondents in the low SES group. Increases in ownership of ITNs occurred among medium-low, medium and high SES groups. The increases in ownership and use of ITNs among medium and high SES groups were, in particular, larger and occurred even though the increase in access to nets was substantially higher in the low SES group. Study findings indicate that the price of nets was not low enough to encourage purchase of nets among the poorest rural respondents: about 92% of respondents who did not use a net reported the price as a reason for non-use. This suggests that net prices may have to be kept extremely low in order for the poorest households in rural Zambia to purchase nets.

This study provides a useful empirical illustration of how changes in SES inequities in knowledge and use of nets are effected by an ITN intervention that reaches socio-economic groups differentially. Declines in SES inequities in knowledge of nets and treatment occurred even though increases in these indicators were larger among medium-low, medium and high SES groups. Similarly, SES inequities in ITN purchase and ownership declined as there was (some) change among medium-low and (larger) change among medium and high SES groups. These findings indicate that SES inequities are likely to decline if, at a minimum, medium-low and medium SES groups are reached through an ITN intervention. Overall, these findings are consistent with previous studies that suggest that it may be difficult for social-marketing ITN interventions to reach the lowest SES groups in rural areas of Africa unless the price of nets is kept very low. More research is needed on the optimal pricing of ITNs for rural areas in Africa.

## Authors' contributions

SA designed the study, conducted the literature review, interpreted the results and wrote the report. RVR conducted the statistical analysis and wrote up the results. GS conceptualized the study. TK designed the questionnaire and implemented the survey.

## References

[B1] Korenromp LE, Miller J, Cibulskis RE, Cham MK, Alnwick D, Dye C (2003). Monitoring mosquito net coverage for malaria control in Africa: possession vs. use by children under 5 years. Trop Med Int Health.

[B2] Worrall E, Hill J, Webster J, Mortimer J (2005). Experience of targeting subsidies on insecticide-treated nets: what do we know and what are the knowledge gaps?. Trop Med Int Health.

[B3] Snow RW, McCabe E, Mbogo CNM, Molyneux CS, Some ES, Mung'ala V, Nevill CG (1999). The effect of delivery mechanism on the uptake of bed net re-impregnation in Kilifi District, Kenya. Health Policy Plan.

[B4] Kolaczinski J, Hanson K (2006). Costing the distribution of insecticide-treated nets: a review of cost and cost-effectiveness studies to provide guidance on standardization of costing methodology. Malar J.

[B5] Onwujekwe O, Hanson K, Fox-Rushby J (2004). Inequalities in purchase of mosquito nets and willingness to pay for insecticide-treated nets in Nigeria: Challenges for malaria control interventions. Malar J.

[B6] Onwujekwe O, Malik EM, Mustafa SH, Mwanza A (2005). Socio-economic inequity in demand for insecticide-treated nets, in-door residual house spraying, larviciding and fogging in Sudan. Malar J.

[B7] Worrall E, Basu S, Hanson K (2002). Is malaria disease of poverty? A review of the literature. Trop Med Int Health.

[B8] Noor AM, Omumbo JA, Amin AA, Zurovac D, Snow RW (2006). Wealth, mother's education and physical access as determinants of retail sector net use in rural Kenya. Malar J.

[B9] Holtz TH, Marum LH, Mkandala C, Chizani N, Roberts JM, Macheso A, Parise ME, Kachur P (2002). Insecticide-treated bednet use, anaemia, and malaria parasitaemia in Blantyre District, Malawi. Trop Med Int Health.

[B10] Stevens W, Wiseman V, Ortiz J, Chavasse D (2005). The costs and effects of a nationwide insecticide treated net programme: the case of Malawi. Malar J.

[B11] National Statistical Office [Malawi] and ORC Macro (2001). Malawi Demographic and Health Survey 2000.

[B12] National Statistical Office [Malawi] and ORC Macro (2005). Malawi Demographic and Health Survey 2004.

[B13] Nathan R, Masanje H, Mshinda H, Schellenberg JA, de Savigny D, Lengeler C, Tanner M, Victora CG, Armstrong Schellenberg JRM (2004). Mosquito nets and the poor: can social marketing redress inequities in access?. Trop Med Int Health.

[B14] Central Statistical Office [Zambia] (1997). Zambia Demographic and Health Survey 1996.

[B15] National Malaria Control Center (2000). Malaria in Zambia. Situation Analysis.

[B16] Baume C, Helitzer D, Kachur P (2000). Patterns of care for childhood malaria in Zambia. Soc Sci Med.

[B17] Shelley K (1998). Formative research on malaria prevention and mosquito net use, Eastern Province, Zambia.

[B18] Central Statistical Office [Zambia] (2001). 2000 Census of Population and Housing, Republic of Zambia.

[B19] Wagstaff A, Paci P, van Doorsaler E (1991). On the measurement of inequalities in health. Soc Sci Med.

